# Navigating the Complexity of Scoring Systems in Sepsis Management: A Comprehensive Review

**DOI:** 10.7759/cureus.54030

**Published:** 2024-02-11

**Authors:** Venkat Reddy, Harshitha Reddy, Rinkle Gemnani, Sunil Kumar, Sourya Acharya

**Affiliations:** 1 Department of General Medicine, Jawaharlal Nehru Medical College, Datta Meghe Institute of Higher Education and Research, Wardha, IND

**Keywords:** qsofa, apache, sofa, sepsis management, scoring systems, sepsis

## Abstract

This comprehensive review navigates the intricate landscape of sepsis scoring systems, aiming to provide healthcare professionals and researchers with a nuanced understanding of their role in contemporary sepsis management. Beginning with a succinct overview of sepsis, the review emphasizes the significance of scoring systems in standardizing assessments and guiding clinical decision-making. Through a detailed analysis of prominent systems such as SOFA, APACHE, and qSOFA, the review delineates their unique attributes, strengths, and limitations. The implications for sepsis management and patient outcomes are discussed, highlighting the potential for these tools to enhance early detection and intervention. The review concludes with a compelling call to action, urging healthcare professionals to integrate scoring systems into routine practice and researchers to explore novel approaches. By synthesizing current knowledge and addressing future directions, this review serves as a valuable resource for those seeking clarity and guidance in the dynamic landscape of sepsis management.

## Introduction and background

Sepsis, often referred to as a "silent killer," arises when the body's immune response to infection becomes dysregulated, leading to systemic inflammation and organ dysfunction. The condition can progress rapidly, evolving into severe sepsis and septic shock if not promptly recognized and treated. Despite medical advancements, sepsis remains a leading cause of morbidity and mortality globally, underscoring the urgency of effective management strategies [1]. The complexity of sepsis necessitates systematic approaches for evaluating its severity and predicting patient outcomes. Scoring systems have emerged as indispensable tools in managing sepsis, offering healthcare professionals structured frameworks to assess organ dysfunction, guide treatment decisions, and gauge the overall prognosis [2].

The significance of scoring systems in sepsis management highlights their role in standardizing the evaluation process, facilitating communication among healthcare providers, and aiding in risk stratification. By providing a standardized language and framework, scoring systems contribute to more consistent and evidence-based clinical decision-making, ultimately improving patient care [3]. Considering the diverse scoring systems available for sepsis management, this comprehensive review aims to navigate the complexity of these tools. The purpose is to offer healthcare professionals, researchers, and educators a detailed exploration of existing scoring systems, their strengths, limitations, and comparative effectiveness. By synthesizing current knowledge, this review seeks to empower clinicians to make informed decisions in the dynamic landscape of sepsis management. Through critical analysis and synthesis of available literature, the review will contribute to a better understanding of the practical implications, challenges, and future directions in applying sepsis scoring systems. As sepsis management continues to evolve, this review aims to be a valuable resource for those seeking clarity and guidance in utilizing scoring systems for optimal patient outcomes.

## Review

Understanding sepsis scoring systems

Sequential Organ Failure Assessment (SOFA) Score

Components and calculation: The SOFA score is a widely utilized scoring system within adult intensive care settings to quantitatively depict organ failures' extent and intensity in sepsis patients [4,5]. The SOFA score encompasses six distinct evaluations, one for each of the respiratory, cardiovascular, hepatic, coagulation, renal, and neurological (central nervous system) systems [6]. Each organ system is assigned a score ranging from 0 (indicating normal function) to 4 (indicating the most abnormal function), resulting in a minimum SOFA score of 0 and a maximum of 24 [6]. A higher SOFA score is indicative of a more severe sepsis-related organ dysfunction [7]. The development of the SOFA score was guided by principles aimed at ensuring ease of use. Organ dysfunction or failure is perceived as a dynamic process rather than a singular event, and as such, it should not be categorized simply as 'present' or 'absent.' Given the rapid fluctuations in organ function among critically ill patients, the score must be routinely repeated, preferably daily, to capture the evolving nature of the condition over time. To facilitate frequent application, the number of variables in the SOFA score has been intentionally kept low [4]. Extensive validation studies involving large cohorts of critically ill patients have confirmed the SOFA score's efficacy in furnishing valuable prognostic insights into in-hospital survival outcomes [5]. Its widespread adoption extends across diverse medical contexts, including medical, trauma, surgical, cardiac, and neurological intensive care units (ICUs) [6]. Nonetheless, scholarly contention suggests that the SOFA score may not reliably predict mortality in cases of isolated organ failures. Notably, patients experiencing primary respiratory failure tend to generate SOFA scores within the range of 4-6, leading to skepticism about the score's accuracy in such scenarios [8]. The SOFA score is valuable for gauging the severity of sepsis-related organ dysfunction in patients. Its user-friendly design, routine applicability, and robust validation in large patient populations enhance its utility. However, caution is warranted, particularly in isolated organ failures, where its predictive accuracy for mortality may be subject to limitations.

Clinical significance and limitations: The SOFA score is a valuable predictor of outcomes and mortality in patients with severe sepsis. It furnishes crucial prognostic insights into in-hospital survival by systematically quantifying the number and severity of failed organs. Recent estimations revealing a doubling of severe sepsis hospitalizations underscore the critical need for accurate assessment methods in addressing sepsis [5]. Widely applied across various contexts, the SOFA score is essential in risk stratification bedside clinical evaluation and is a defining characteristic of sepsis syndrome [9]. Despite its widespread use, the score's vintage, spanning over 25 years, has prompted scholars to advocate for an update that better aligns with contemporary practices and incorporates new variables [4]. While the SOFA score remains an indispensable tool, it is not without its considerations. Some scholars contend that its efficacy in predicting mortality at 24 hours and across diverse types of infections may be limited. Furthermore, the score's predictive value may exhibit variability when applied in different economic settings, distinguishing between high-income and low- to middle-income countries [10]. Despite these acknowledged limitations, the SOFA score retains its merit as a valuable instrument for assessing the severity of sepsis-related organ dysfunction, boasting high accuracy in delineating the course of organ dysfunction in septic patients [7].

Acute Physiology and Chronic Health Evaluation (APACHE) Score

Overview of APACHE II and APACHE III: The APACHE score is a widely employed illness severity metric in critical care medicine designed to forecast mortality. Among its various iterations, APACHE II and APACHE III are prominent. The APACHE II score is derived from the assessment of 12 physiological variables, measured within 24 hours of admission, in conjunction with the patient's age and pre-existing health status. These variables encompass AaDO2 or PaO2, body temperature, mean arterial pressure, blood pH, heart rate, respiratory rate, serum sodium, and serum potassium [11]. Utilized to provide insight into a patient's morbidity and facilitate outcome comparisons with peers, the APACHE II score serves as a valuable tool for estimating mortality in critically ill individuals [12]. As a general gauge of disease severity, it integrates current physiological measurements, age, and the individual's health history [12]. An essential caveat is that the APACHE II score has not undergone validation for application in the pediatric population or individuals under the age of 16 [12]. Therefore, its utility in assessing illness severity and predicting outcomes in children or young people remains unverified.

Application in sepsis management: The APACHE score, particularly APACHE III, has become integral in the realm of sepsis management for predicting patient outcomes and evaluating illness severity. Notably, studies have delved into the comparative effectiveness of APACHE III and other scoring systems, such as Simplified Acute Physiology Score II (SAPS II), in forecasting sepsis outcomes within ICUs [1]. The distinct predictive capabilities of APACHE III and SAPS II have been subject to scrutiny, revealing that neither system unequivocally outperforms the other in terms of accuracy [13]. APACHE III emerges as a significant determinant in forecasting patient outcomes within the framework of sepsis management [13]. The subsequent iteration, APACHE IV, has undergone validation for its ability to predict the length of stay (LOS) in the ICU for patients grappling with sepsis [14]. However, findings from a study indicate that APACHE IV tends to overpredict ICU LOS in septic patients, revealing suboptimal performance in this predictive aspect [14]. While APACHE scores, especially APACHE III, have proven useful in sepsis management for prognostication and severity assessment, it is imperative to acknowledge the limitations inherent in these scoring systems. The study's revelation regarding the overprediction of ICU LOS by APACHE IV underscores the ongoing need for refinement in subsequent versions to enhance their predictive capabilities. As the landscape of critical care evolves, continual improvements and adaptations to scoring systems may be crucial for maintaining their effectiveness in guiding clinical decisions.

Quick Sepsis-Related Organ Failure Assessment (qSOFA)

Development and simplicity: The qSOFA score is a bedside prompt used to identify patients with suspected infection at greater risk for a poor outcome outside the ICU. It uses three criteria, assigning one point for low blood pressure (SBP≤100 mmHg), high respiratory rate (≥22 breaths per min), or altered mentation (Glasgow Coma Scale<15) [15]. The qSOFA score has the advantage of simplicity, making it a valuable tool for quickly assessing patients at risk of sepsis-related complications [16]. However, some studies have suggested that qSOFA may not be sensitive enough to predict 28-day mortality in emergency department patients with sepsis [17]. Despite its simplicity, the predictive validity of qSOFA for in-hospital mortality has been debated, with some studies indicating modestly better prognostic accuracy than other scoring systems [18]. Therefore, while qSOFA is a simple and convenient tool, its effectiveness in predicting mortality and clinical deterioration in sepsis patients may vary. It should be interpreted in the context of other clinical indicators and scoring systems [19].

Clinical utility and controversies: The qSOFA score has been widely used as a bedside tool to identify patients with suspected infection who are at greater risk for a poor outcome outside the ICU. The qSOFA score is simple and easy to use, but some studies have suggested that it may not be sensitive enough to predict 28-day mortality in emergency department patients with sepsis [17]. Other studies have found that qSOFA has modest prognostic accuracy in predicting in-hospital mortality compared to other scoring systems [20]. However, qSOFA is useful in detecting clinical deterioration in infected patients outside the ICU [16]. Despite its clinical utility, there are controversies surrounding using qSOFA, particularly in predicting mortality in sepsis patients. Therefore, qSOFA should be interpreted in the context of other clinical indicators and scoring systems, and experienced clinical judgment should always be pre-eminent [15].

Comparative analysis of scoring systems

SOFA vs. APACHE: Strengths and Weaknesses

The APACHE and SOFA scoring systems are commonly used in intensive care to assess the severity of illness and predict patient outcomes.

APACHE

Strengths: To predict mortality, consider physiological variables, chronic health conditions, and admission type. Can be used to calculate standardized mortality ratios for large patient populations [21].

Weaknesses: Scoring is based on the most abnormal measurements in the first 24 hours of ICU stay, which may not reflect the patient's condition over time. Higher scores are generally associated with worse outcomes, but it does not provide a predicted mortality algorithm [21].

SOFA

Strengths: Provides a defined score for each of the six organ systems, allowing for daily scoring during the course of the ICU stay. Higher SOFA scores are associated with worse outcomes, making it a useful tool for assessing organ dysfunction [21].

Weaknesses: Does not have a predicted mortality algorithm is not designed to predict mortality for large patient populations [21]. The scoring duration is not standardized, as it allows for daily scoring during the ICU stay, which may introduce variability in the assessment [21].

A comparative analysis of the performance of APACHE II, SOFA, and modified Nutrition Risk in the Critically Ill (mNUTRIC) scoring systems in critically ill patients found that APACHE II and SOFA had better sensitivity and specificity than the mNUTRIC score. However, the mNUTRIC score was more sensitive in predicting outcomes related to the need for mechanical ventilation [22]. In a systemic review of different ICU scoring systems, including SOFA, SAPS II, APACHE II, and APACHE III, the APACHE systems were slightly superior to the SAPS II and SOFA in predicting mortality [23]. Both the APACHE and SOFA scoring systems have their strengths and weaknesses. While APACHE is useful for predicting mortality in large patient populations, SOFA is valuable for assessing organ dysfunction and can be scored daily during the ICU stay. The choice of which system to use may depend on the specific clinical context and the outcomes of interest.

qSOFA vs. SOFA: Practical Implications

The qSOFA and SOFA scores are used to assess the severity of sepsis and predict patient outcomes. The qSOFA score is used to identify patients with suspected infection at high risk for in-hospital mortality outside of the ICU setting. On the other hand, the SOFA score predicts mortality risk for patients in the ICU based on lab results and clinical data. A study comparing the performance of qSOFA and SOFA scores in predicting in-hospital mortality among adult critical care patients with suspected infection found that a score of two or more is better than a qSOFA score in predicting in-hospital mortality [24]. Another study found that for patients outside of the ICU with a qSOFA score ≥ 2, there was a 3- to 14-fold increase in the rate of in-hospital mortality. Among ICU patients, however, the predictive validity of the SOFA for in-hospital mortality was statistically greater than the qSOFA [25]. The qSOFA score is presented only as an additional clinical criterion in identifying suspected sepsis, and the Sepsis-3 task force recommended that a positive qSOFA score should prompt the calculation of a SOFA score to confirm the diagnosis of sepsis. However, this recommendation remains controversial, as the qSOFA is more predictive than the SOFA outside the ICU setting [25]. The qSOFA and SOFA scores have different practical implications. The qSOFA score is useful for identifying patients with suspected infection at high risk for in-hospital mortality outside of the ICU setting. In contrast, the SOFA score is useful for predicting mortality risk for patients in the ICU based on lab results and clinical data. The choice of score to use may depend on the specific clinical context and the outcomes of interest. Table 1 lays out the advantages and disadvantages of the scoring system below:

**Table 1:** Advantages and disadvantages of the scoring system SOFA: Sequential Organ Failure Assessment; APACHE: Acute Physiology and Chronic Health Evaluation, qSOFA: Quick Sepsis-related Organ Failure Assessment, ICU: intensive care unit; mNUTRIC: modified Nutrition Risk in the Critically Ill

Scoring System	Advantages	Disadvantages
SOFA	Comprehensive assessment of multiple organ systems	Complexity may limit real-time applicability
	Widely accepted in research and clinical settings	Resource-intensive, requiring extensive lab data
	Utilizes objective criteria, reducing subjectivity	May not be as sensitive for early sepsis detection
APACHE	Aids in risk stratification	Resource-intensive, requiring detailed clinical data
	Extensively validated and widely used in ICUs	Scoring variability due to subjective components
	Incorporates pre-existing health conditions	Originally designed for general ICU populations
qSOFA	Simple and easy to use at the bedside	Limited comprehensive assessment of organ dysfunction
	Focuses on three readily available clinical parameters	May lack specificity for sepsis
	Designed for early identification of high-risk patients	Not suitable for all clinical settings
Goal score	Individualized targets based on patient response	Subjectivity in goal setting may introduce variability
	Applicable to various clinical scenarios	Requires meticulous documentation for clarity
	Offers flexibility in goal-setting	Implementation may demand additional resources
mNUTRIC Score	Focuses on nutritional status	Limited scope, primarily nutritional assessment
	Utilizes objective nutritional parameters	Dependence on nutritional data availability
	Can contribute to early intervention for nutritional care	May not provide a comprehensive evaluation of sepsis

APACHE II Converted to APACHE III and APACHE IV

APACHE scoring system has evolved from APACHE II to APACHE IV. APACHE II was published in 1985, while APACHE IV, the latest version, was published in 2006. APACHE IV is recommended instead of APACHE II and III, as it is built on the study of a more recent patient population and standard of care. It has a more complex scoring system, considering more variables and providing an estimated risk of death and length of stay. APACHE III and IV are very similar, using the same variables, with only the disease-specific coefficients being updated. Studies have compared the predictive abilities of APACHE II and APACHE IV, with some showing that both scoring systems work equally well. In contrast, others have demonstrated APACHE IV as a better predictor of mortality than APACHE II. Some studies found APACHE IV has better discrimination but poor calibration compared to APACHE II [26-29].

Considerations for Choosing the Appropriate Score

Various critical factors must be carefully weighed when selecting the most suitable scoring system. The decision-making process is intricately tied to an understanding of the specific clinical context, the desired outcomes, and the availability of resources. In delineating the clinical Context, it is crucial to identify the precise medical scenario in which the scoring system will find application. For example, the qSOFA and SOFA scores prove instrumental in evaluating sepsis severity. In contrast, the APACHE and SOFA scores are tailored for assessing the severity of illness and predicting patient outcomes within intensive care settings [22]. Identifying the outcomes of interest is paramount, focusing on mortality, morbidity, or the necessity for specific interventions. Careful consideration of these outcomes assists in selecting a scoring system that closely aligns with the desired clinical objectives [30]. The availability of resources is a practical consideration that underscores the need to assess the accessibility of essential tools such as laboratory tests, imaging studies, and diagnostic tools. This evaluation is crucial, as certain scoring systems may demand resources that could be limiting in diverse healthcare settings [31].

Opting for a scoring system characterized by simplicity and ease of use holds significance in enhancing compliance and accuracy in routine clinical practice. A user-friendly system contributes to more seamless integration into daily healthcare workflows. Ensuring the validity and reliability of the chosen scoring system is paramount. The system should have a robust foundation of validation within the target population, coupled with demonstrated reliability and predictive power [30]. Compatibility with existing systems is another aspect that warrants attention. Careful consideration should be given to how well the selected scoring system integrates with pre-existing systems, evaluating whether its incorporation enhances existing processes and adds value to overall clinical workflows. Recognizing the dynamic nature of medical knowledge and evolving clinical practices, it is imperative to acknowledge that scoring systems may require continuous improvement. Regular reviews of the chosen scoring system and a willingness to explore alternatives when necessary are vital components of a commitment to continuous enhancement. In essence, the judicious selection of an appropriate scoring system hinges on a nuanced evaluation of the specific clinical context, the outcomes of interest, resource availability, simplicity and ease of use, validity, and reliability, compatibility with existing systems, and a steadfast commitment to continuous improvement [31].

Evolving trends in sepsis scoring

Newer Scoring Systems and Research Developments

mNEWS: One noteworthy development is the emergence of mNEWS (modified National Early Warning Score), as highlighted in a comparative study of sepsis scoring systems. mNEWS demonstrated exceptional sensitivity (96.48%) and negative predictive value (80%) in predicting sepsis among patients presenting to the emergency department with suspected infection [32]. In the same study, mNEWS also displayed the highest area under the receiver operating characteristic curve (AUC) for predicting sepsis, with an AUC of 0.685 [32].

qSOFA: The qSOFA system has undergone scrutiny compared to other scoring systems, such as SIRS and NEWS, particularly in predicting mortality and sepsis in emergency department patients. Notably, qSOFA exhibited the highest specificity (69.49%) and positive predictive value (65.05%) for predicting sepsis [32].

CEC SEPSIS KILLS pathway: In a comprehensive study evaluating diverse scoring systems and pathways, the CEC SEPSIS KILLS pathway, incorporating the modified Shapiro rule, demonstrated notable sensitivity (88.05%) in detecting bacteremia within the emergency department population [33].

Investigation into biomarkers: Researchers are actively investigating clinically relevant biomarkers as potential tools for improving the accuracy of sepsis diagnosis. While ongoing, definitive outcomes from these investigations are yet to be realized [34].

Precision medicine techniques and targeted therapy: Advancements in precision medicine techniques and the development of targeted therapies tailored to sepsis management represent promising avenues for improving patient outcomes. The application of precision medicine principles holds the potential to enhance the effectiveness of interventions in sepsis cases [34]. It is imperative to emphasize that, despite these advancements, no scoring system can fully substitute the nuanced judgment of experienced clinicians. The deployment of scoring criteria in emergency departments poses challenges, and ongoing research and development remain pivotal to refining sepsis scoring systems and diagnostic methods. The ultimate goal is to enhance early identification and treatment of sepsis, thereby improving patient outcomes and reducing the burden of this critical condition [35].

Integration of Biomarkers and Advanced Diagnostics

Biomarkers are a valuable tool for early diagnosis, identifying patients at high risk of complications, and monitoring disease progression in sepsis. Several biomarkers have been identified, including C-reactive protein (CRP), procalcitonin (PCT), and CD64 index. However, no single biomarker is likely to adequately reflect the rapidly evolving nature of a potentially septic patient's condition, and a multi-marker approach may be more effective. A combination of pro- and anti-inflammatory biomarkers in a multi-marker panel may help identify patients at risk of sepsis and improve the accuracy of sepsis diagnosis. Investigation into clinically relevant biomarkers of sepsis is ongoing, and recent advances have led to the development of newly identified classes of biomarkers, such as long-non-coding RNAs and the human microbiome. However, no effective results have been yielded yet. Advances in precision medicine techniques and targeted therapy directed at sepsis management are expected to improve patient outcomes [34,36-39].

Artificial Intelligence and Machine Learning in Sepsis Prediction

Artificial intelligence (AI) and machine learning (ML) are increasingly used in sepsis prediction and management. Recent developments include the creation of AI algorithms such as "Sepsis Early Risk Assessment (SERA)" and "InSight scores," which utilize clinical data from electronic health records (EHR) to predict and diagnose sepsis [40-42]. These AI-derived algorithms have shown promise in early prediction, prognosis assessment, mortality prediction, and optimal management of sepsis [43]. Empirical studies have demonstrated the potential of deployed sepsis prediction algorithms to improve care and reduce mortality, highlighting the value of AI in sepsis management [44]. However, the real-world integration of AI algorithms for sepsis prediction is still under development, and further research is needed to assess their clinical effectiveness and application in healthcare settings [41]. AI and ML technologies are potentially revolutionizing sepsis prediction and management, offering opportunities to enhance early detection, diagnosis, and treatment of this life-threatening condition. However, ongoing research and real-world implementation are essential to fully realize the benefits of these advanced technologies in clinical practice.

Clinical applications and implementation challenges

Use of Scores in Early Sepsis Detection

The use of sepsis scoring criteria in early sepsis detection presents challenges, as these criteria are not always available in a busy emergency department (ED), which can hinder the predictive accuracy of scoring systems and indirectly contribute to patient outcomes [35]. Several scoring systems, such as SIRS, NEWS, qSOFA, and SOFA, have been compared regarding their accuracy for sepsis detection and mortality prediction [45]. It is important to note that no single marker or physiologic parameter consistently predicts the imminent development of sepsis [46]. Additionally, the absence of certain scoring criteria should not prevent clinicians from engaging in prompt investigation and management of sepsis, as experienced clinical judgment is always crucial [3]. Therefore, while these scoring systems can be useful for specific purposes, they should be complemented with clinical assessment and prompt management to ensure accurate diagnosis and timely intervention [3,35].

Impact on Decision-Making in Different Healthcare Settings

The use of sepsis scoring criteria in early sepsis detection can significantly impact decision-making in different healthcare settings. Automated systems that continuously monitor patient status, such as vital signs, and alert clinicians if criteria for possible sepsis are met have been shown to increase survival by prompting quick initiation of treatment [47]. However, the availability and accuracy of scoring criteria can vary, with some studies highlighting the need for more research to determine the optimal variables and thresholds for sepsis screening, especially in the prehospital setting [35,48]. Additionally, the broad adoption of sepsis screening tools or advanced surveillance/detection/alerting systems remains a challenge, and the diagnostic utility of these tools may suffer when applied to different areas due to variations in patient populations and care settings [49]. Therefore, while sepsis scoring systems can be valuable in early detection, they should be complemented with clinical assessment and prompt management to ensure accurate diagnosis and timely intervention [47,49].

Barriers to Effective Implementation in Clinical Practice

Barriers to effectively implementing sepsis scoring criteria in clinical practice span several dimensions, encompassing individual, health system, and contextual factors. One major hindrance is the lack of access, where healthcare professionals may find themselves without the necessary resources or guidelines to implement sepsis scoring systems [50] effectively. This limitation could impede the seamless integration of scoring criteria into routine clinical assessments. Another substantial challenge arises from the complexity of guideline documents, often characterized by many weak or conditional recommendations. This complexity can make it difficult for healthcare professionals to navigate and apply the guidelines in their day-to-day practice [51].

Time constraints emerge as a significant barrier, with healthcare professionals facing challenges due to their demanding clinical responsibilities. The limited time available for research, study, and guideline implementation poses a notable challenge to incorporating sepsis scoring systems into routine clinical workflows [51,52]. Moreover, a lack of knowledge and skills presents a formidable obstacle. Healthcare professionals may find themselves without the necessary expertise to effectively implement sepsis scoring systems and may lack a comprehensive understanding of the benefits associated with their use [52,53]. The poor applicability of some guidelines in real-world practice poses another challenge for healthcare professionals. When guidelines do not align with the complexities of clinical scenarios, their implementation becomes impractical and challenging [53].

In addition, suboptimal healthcare networks and interprofessional communication pathways can hinder the effective implementation of guidelines. Poor communication and coordination among healthcare teams may impede the seamless integration of sepsis scoring systems into collaborative care efforts [53]. The motivational aspect also comes into play, as lack of motivation and adherence among healthcare professionals can be influenced by various factors such as resource constraints, time limitations, or perceived ineffectiveness of the scoring systems [53]. Furthermore, inadequate reinforcement or support from healthcare organizations and institutions adds to the list of barriers. Without sufficient backing, healthcare professionals may face difficulties in implementing sepsis scoring criteria effectively [53]. To overcome these multifaceted barriers, addressing underlying issues is paramount. Providing healthcare professionals with the necessary resources, training, and support is a crucial strategy to facilitate the seamless integration of sepsis scoring criteria into clinical practice.

Critique and future directions

Limitations and Criticisms of Existing Scoring Systems

The existing sepsis scoring systems, such as qSOFA, SIRS, EWS, and SOFA, have been subject to limitations and criticisms. It is recognized that no scoring system can serve as a stand-alone definition of sepsis, and the absence of specific criteria should not hinder clinicians from promptly investigating and managing sepsis [3]. The recently updated definitions of sepsis have shifted focus from inflammation to life-threatening organ dysfunction caused by a dysregulated host response [3]. While these scoring systems can be valuable for specific purposes, they should be complemented with clinical assessment and prompt management to ensure accurate diagnosis and timely intervention [3]. Several studies have compared the performance of different scoring systems. For instance, one study found that mNEWS had the highest sensitivity and negative predictive value for predicting sepsis. In contrast, qSOFA had the highest specificity and positive predictive value [32]. Another study highlighted the poor sensitivity of the qSOFA in identifying mortality risk or the likelihood of requiring ICU, suggesting the need for re-evaluation of its clinical usefulness [33]. While sepsis scoring systems can provide valuable information, they have limitations and should be used with clinical judgment and prompt management. The evolving understanding of sepsis and the need for accurate and timely diagnosis warrant continued research and potential refinements to existing scoring systems.

Emerging Trends and Innovations in Sepsis Scoring

The existing sepsis scoring systems, such as qSOFA, SIRS, EWS, and SOFA, have been subject to limitations and criticisms. It is recognized that no scoring system can serve as a stand-alone definition of sepsis, and the absence of specific criteria should not hinder clinicians from promptly investigating and managing sepsis [3,35]. The recently updated definitions of sepsis have shifted focus from inflammation to life-threatening organ dysfunction caused by a dysregulated host response [3]. While these scoring systems can be valuable for specific purposes, they should be complemented with clinical assessment and prompt management to ensure accurate diagnosis and timely intervention [3,35]. Several studies have compared the performance of different scoring systems. For instance, one study found that mNEWS had the highest sensitivity and negative predictive value for predicting sepsis. In contrast, qSOFA had the highest specificity and positive predictive value [54]. Another study highlighted the poor sensitivity of the qSOFA in identifying mortality risk or the likelihood of requiring ICU, suggesting the need for re-evaluation of its clinical usefulness [46]. While sepsis scoring systems can provide valuable information, they have limitations and should be used with clinical judgment and prompt management. The evolving understanding of sepsis and the need for accurate and timely diagnosis warrant continued research and potential refinements to existing scoring systems.

Recommendations for Future Research and Development

The key challenges in surviving sepsis revolve around early diagnosis and treatment, emphasizing the need for continued research efforts. Investigating methods to stratify patients using biomarkers and other tools holds promise in enhancing the speed of diagnosis and treatment, a critical factor in improving sepsis outcomes [55]. The landscape of sepsis treatment is expected to evolve towards more individualized approaches over the next five years. This transformation comprehensively considers factors such as the individual's profile, type of infection, chronic conditions, and overall patient characteristics, contributing to a more tailored and effective treatment paradigm [55].

Biomarkers and predictive analytics represent another avenue of exploration for researchers in understanding the pathophysiological aspects and potential management strategies of sepsis. Ongoing studies aim to refine diagnostic and prognostic capabilities, improving the overall understanding and treatment of sepsis [55]. The future of sepsis treatment should also delve into the intricate relationship between microbiomes and immunomodulation in developing and progressing sepsis. Exploring these factors holds the potential to uncover novel therapeutic interventions that could revolutionize sepsis management [55]. In diagnostics, the development of new tools, including artificial intelligence, emerges as a critical component for achieving better, less invasive, and more accurate sepsis diagnoses. Incorporating advanced technologies into diagnostic processes is essential for enhancing the efficiency and precision of early identification and intervention [55].

Effectively combating antimicrobial resistance and improving patient outcomes in sepsis management necessitates the development of robust antimicrobial strategies. Research efforts should be directed toward discovering innovative approaches to address this pressing concern and ensure the efficacy of sepsis treatments [55]. Furthermore, addressing the challenge of missing scoring criteria in emergency departments is crucial. Researchers should explore strategies to enhance the completeness and accuracy of scoring systems, which play a vital role in predicting outcomes and guiding patient management [35]. By concentrating on these critical areas, researchers have the potential to significantly advance the early diagnosis, treatment, and overall management of sepsis, ultimately leading to improved patient outcomes.

## Conclusions

In conclusion, this comprehensive review underscores the critical role of sepsis scoring systems in shaping contemporary approaches to sepsis management. The analysis of prominent scoring tools, including SOFA, APACHE, and qSOFA, has revealed nuanced insights into their respective strengths and limitations, providing clinicians with a foundation for informed decision-making. The implications for sepsis management are profound, as the judicious application of these scoring systems can significantly impact early sepsis recognition, guide interventions, and ultimately contribute to improved patient outcomes. The dynamic nature of sepsis management is emphasized, urging healthcare professionals to integrate these tools into routine clinical practice and stay abreast of emerging trends and technologies. The call to action extends to both healthcare professionals and researchers, encouraging collaborative efforts to enhance proficiency in scoring system utilization, validate existing models, and explore innovative approaches. By heeding this call, the healthcare community can collectively advance sepsis management strategies, fostering a future where timely and precise interventions mitigate the impact of this life-threatening condition on global health.
